# Total CroFab and Anavip Antivenom Vial Administration in US Rattlesnake Envenomations: 2019–2021

**DOI:** 10.1007/s13181-023-00941-7

**Published:** 2023-04-28

**Authors:** Nicklaus Brandehoff, Alicia Dalton, Claire Daugherty, Richard C. Dart, Andrew A. Monte, On behalf of the ToxIC Snakebite Study Group

**Affiliations:** 1 Rocky Mountain Poison & Drug Center, Denver Health and Hospital Authority, Denver, CO USA; 2 Department of Emergency Medicine, University of Colorado School of Medicine, Aurora, CO USA; 3 Asclepius Snakebite Foundation, Seattle, WA USA; 4 Claire Daugherty Consulting LLC, Salt Lake City, UT USA

**Keywords:** Rattlesnake, Antivenom, CroFab, Anavip, Fab, Fab2

## Abstract

**Introduction:**

In 2018, Anavip became available for the treatment of rattlesnake envenomations in the USA. No comparisons between the treatment characteristics of patients have been made since Anavip and CroFab have both been widely available. The objective of this study was to compare the number of antivenom vials administered of CroFab and Anavip during the treatment of rattlesnake envenomations in the USA.

**Methods:**

This was a secondary analysis of rattlesnake envenomations utilizing the North American Snakebite Registry (NASBR) from 2019 through 2021. Frequencies and proportions were used to summarize demographics and baseline clinical characteristics. The primary outcome was total antivenom vials administered during treatment. Secondary outcomes included the number antivenom administration events, total treatment time, and hospital length of stay.

**Results:**

Two hundred ninety-one rattlesnake envenomations were analyzed; most occurred in the Western USA (*n* = 279, 96 %). One hundred one patients (35%) received only CroFab, 110 (38%) received Anavip only, and 80 (27%) received both products. The median number of vials used was 10 for CroFab, 18 for Anavip, and 20 for both antivenoms. More than one antivenom administration was necessary in thirty-nine (39%) patients that received only CroFab and 76 (69%) patients that received Anavip only. The median total treatment time was 5.5 hours for CroFab, 6.5 for Anavip, and 15.5 hours when both antivenoms were administered. All antivenom groups had a median hospital length of stay of 2 days.

**Conclusions:**

Rattlesnake envenomated patients in the Western USA treated with CroFab had fewer antivenom vials and fewer antivenom administrations compared to patients treated with Anavip.

**Supplementary Information:**

The online version contains supplementary material available at 10.1007/s13181-023-00941-7.

## Introduction

There are approximately 10,000 venomous snakebites in the USA each year. Ninety-nine percent of envenomations occur due to *Crotalus* or *Sistrurus* species (rattlesnakes) and *Agkistrodon* species (i.e., copperheads and cottonmouths), collectively known as pit vipers. Envenomations from these species can result in local symptoms such as pain, swelling, necrosis, or systemic effects such as coagulation abnormalities, bleeding, paralysis, shock or rarely, death [1, 2].

Antivenoms are the mainstay of treatment for snake envenomations. Before the year 2000, Wyeth Polyvalent antivenom was the only available antivenom to treat pit viper envenomations in the USA. The Wyeth product is a whole IgG derived from horse serum using the venom from Central and North American snakes, Fer-de-lance (*Bothrops atrox*), South American rattlesnake (*Crotalus durissis terrificus*), Eastern Diamondback (*Crotalus adamanteus*), and Western Diamondback (*Crotalus atrox*). It was effective for most pit viper bites in the USA but also carried a significant risk of adverse reactions (23–56%), including a significant rate of anaphylaxis [3].

In 2000, *Crotalidae* Polyvalent Immune Fab (CroFab, BTG International Inc.) was approved by the US Food and Drug Administration (FDA) for treating pit viper envenomations. The CroFab product uses venoms from snakes native to the USA (*C. adamanteus*, *C. atrox*, Mojave green or *C. scutulatus*, and Cottonmouth or *A. piscivorus*), and the included immunoglobulins are derived from sheep. The Fc portion of the IgG is cleaved with the enzyme papain, which yields isolated Fab fragments [4]. Removing the Fc region, along with other purification techniques, results in the development of an effective antivenom with very few adverse reactions compared to the previous Wyeth product (0.6–6% vs. 23–56%) [4, 5]. Wyeth ceased production in 2000, supply waned over the ensuing years, and the safer and effective CroFab antivenom quickly replaced the Wyeth in clinical practice. CroFab is administered as an initial loading bolus of 4–6 vials for initial control (to be re-dosed if venom effect control is not achieved), followed by three two-vial maintenance doses.

In 2018, *Crotalidae* immune F(ab’)2 (Anavip, Instituto Bioclon) was made commercially available initially for rattlesnake envenomation, followed by approval in 2021 for use for *Agkistrodon* envenomation [6]. This antivenom uses Terciopelo (*B. asper*) and *C. durissis* venom and is derived from horses; the harvested immunoglobulins are digested with pepsin, creating a F(ab’)2 fragment. The initial loading dose is 10 vials, with repeat dosing to achieve control of venom effects. There is no scheduled maintenance dosing recommended. The rates of reported adverse reactions are similar to CroFab [7]. Phase III clinical trial data suggest that late-onset and post-treatment recurrent coagulopathies are reduced with the use of Anavip. It is postulated this effect is likely due to the significantly longer half-life of F(ab’)2 compared to Fab fragments [7], though these findings and this hypothesis have not been confirmed in the post-approval setting. Furthermore, it is unclear if repeat dosing may occur, contrary to recommendations, due to the progression of patient symptoms and failure of this antivenom to adequately match some venom components of US snakes.

With the addition of this new antivenom to the market, it is important to assess the use profile of both antivenoms in clinical practice. To date, there is minimal data on the use of Anavip compared to CroFab. This study aimed to compare the number of antivenom vials administered for rattlesnake envenomations in the USA.

## Methods

### Database, Case Identification, and Data Definitions

This was a secondary analysis of the American College of Medical Toxicology’s (ACMT) Toxicology Investigators Consortium (ToxIC) North American Snakebite Registry (NASBR). The registry is approved by the Western IRB; this analysis included no identifying patient information and thus was deemed exempt from IRB review. This study adheres to the ethical principles for medical research outlined in the Declaration of Helsinki.

**Fig. 1:**
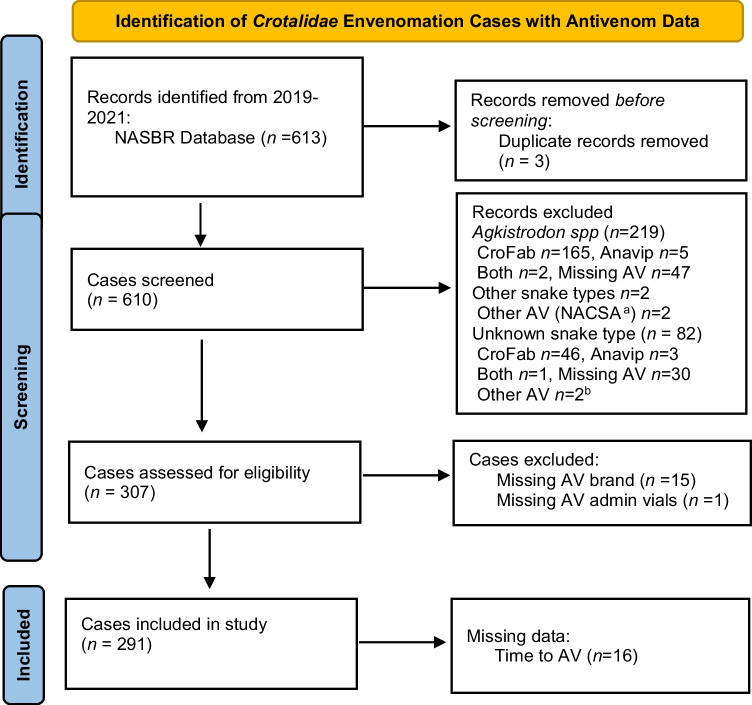
Case identification and exclusion from the NASBR Database, 2019–2021. ^a^NACSA North American Coral Snake Antivenom, ^b^Other antivenom type in cases with unknown snake type—SAIMR Polyvalent, Antivipmyn Tri.

NASBR prospectively collects data about patients treated for snake envenomation from a national network of medical toxicologists who evaluate and treat patients at the bedside. It includes data on demographic and snakebite characteristics, clinical effects of envenomation, and treatment details. We included all rattlesnake cases in the NASBR from 2019–2021. Analyzed cases required complete data on antivenom type and total antivenom vials administered (Fig. 1). Western states were defined as those that do not have native populations of *Agkistrodon* species. Eastern states were those with native populations of *Agkistrodon* species (see Supplemental Appendix). Tissue damage was defined as swelling, necrosis, ecchymosis, erythema, concern for compartment syndrome, or functional deficit present during any of the evaluation or treatment times in the dataset. Upper extremity bites included all bites above the waist, and lower extremity bites included bites below the waist. Coagulopathy included patients that had prothrombin time (PT) > 23 seconds, fibrinogen < 150 mg/dL, or platelet count < 150 K/mm^3^ at any time during their evaluation and treatment. Neurologic toxicity included cases with documented neurotoxicity at initial presentation, as reason for additional antivenom doses or reason for re-administration. Systemic symptoms included any patient with emesis, diarrhea, hypotension, angioedema, or anaphylaxis recorded in the database. Maintenance doses were considered part of the initial antivenom administration for patients receiving CroFab, since this is the approved treatment course. All additional bolus doses and antivenom administered beyond the three scheduled maintenance doses were considered additional administrations. We defined time to antivenom as the time from the envenomation to the time of initial antivenom administration. Total treatment time was defined as the time between the initial antivenom administration and the end of the final administration.

### Analysis

Baseline characteristics, demographics, and antivenom administration details were summarized using descriptive statistics (*n*, median, interquartile range (IQR), for continuous variables and *n*, percent (%) for categorical variables).

The primary outcome was the total number of antivenom vials used. We used an analysis of covariance (ANCOVA) multiple linear regression model, including the categorical variables of age category, sex, bite location, year, region, antivenom type, and the continuous variable, time from the bite to antivenom. This method was used to account for three levels of the primary variable of interest, CroFab, Anavip, and both antivenoms. Additionally, this allowed us to assess the covariates above where simple student *t*-tests or Cochran-Mantel Haenszel tests do not allow for both multi-level categorical analyses with both categorical and continuous covariate assessment. Regression models were initially conducted as univariate, then as a full multivariate, and finally using stepwise selection (with a model factor entry criteria of *p* < 0.15 and a stay criteria of *p* < 0.15). Least squares means and associated standard errors were summarized for the variables included in the regression model after stepwise selection.

Total treatment time, the number of administrations (regardless of the number of vials administered at the timepoint), and hospital length of stay were compared between antivenom types using a nonparametric Kruskal-Wallis test. *P*-values < 0.05 were considered to be statistically significant. No adjustments were made for multiple comparisons. Missing data were not imputed. All analyses were performed using SAS statistical software version 9.4.

## Results

There were 291 rattlesnake envenomations included in this study (Fig. 1). The median age of patients was 32 years old (IQR: 14, 57). Men represented the majority of patients (*n* = 201, 69%), and most were treated in the Western USA (*n* = 279, 96%). The large majority of patients were identified as Caucasian (78%), followed by Hispanic/Latino (18%), Unknown (12%), and Native American (6%). Bites were observed in the lower extremity (*n* = 156, 54%) or upper extremity (*n* = 135, 46%) at similar rates. Tissue damage was the most common clinical effect (*n* = 290, 100%), followed by coagulopathy (*n* = 136, 48%), systemic symptoms (*n* = 52, 18%), and neurologic symptoms (*n* = 34, 12%) (Table 1). There were no deaths recorded.

**Table 1:** Demographics of included cases.

Demographic variable	CroFab *n ***=** 101	Anavip *n* **= 110**	Both antivenoms *n* **= 80**	Overall *N* **= 291**
Median age (IQR)	32.0 (11, 59)	35.5 (14, 56)	31.5 (16, 56)	32.0 (14, 57)
Female sex *n* (%)	33 (32.7)	37 (33.6)	20 (25.0)	90 (30.9)
Race *n* (%)				
Asian African American/Black American Indian/Native American Caucasian/White Native Hawaiian/Pacific Island Unknown Mixed Middle Eastern	2 (2.0) 0 (0) 3 (3.0) 87 (86.1) 0 (0) 7 (6.9) 2 (2.0) 0 (0)	2 (1.8) 3 (2.7) 8 (7.3) 78 (70.9) 1 (0.9) 15 (13.6) 2 (1.8) 1 (0.9)	2 (2.5) 0 (0) 5 (6.3) 61 (76.3) 0 (0) 12 (15.0) 0 (0) 0 (0)	6 (2.1) 3 (1.0) 16 (5.5) 226 (77.7) 1 (0.3) 34 (11.7) 4 (1.4) 1 (0.3)
Ethnicity *n* (%)				
Hispanic/Latino Not Hispanic/Latino	19 (18.8) 82 (81,2)	20 (18.2) 90 (81.8)	12 (15.0) 68 (85.0)	51 (17.5) 240 (82.5)
Region *n* (%)				
East West	12 (11.9) 89 (88.1)	0 (0) 110 (100)	0 (0) 80 (100)	12 (4.1) 279 (95.9)
Envenomation location *n* (%)				
Lower extremity Upper extremity	55 (54.5) 46 (45.5)	63 (57.3) 47 (42.7)	38 (47.5) 42 (52.5)	156 (53.6) 135 (46.4)
Clinical effects *n* (%)				
Tissue damage Coagulopathy Neurologic symptoms Systemic symptoms	101 (100) 48 (47.5) 16 (15.8) 21 (20.8)	109 (99.1) 41 (37.3) 8 (7.3) 15 (13.6)	80 (100) 47 (58.8) 10 (12.5) 16 (20.0)	290 (99.7) 136 (46.7) 34 (11.7) 52 (17.9)
Time to antivenom (hours) (IQR)^a^	2.0 (1.5, 4)	3.0 (1.5, 5)	2.5 (2, 3.5)	2.5 (1.75, 4)
Median number of vials (IQR)	10 (6, 12)	18 (10, 25)	20 (16, 28)	15 (10, 22)
Median number of administrations (IQR)^b^	1 (1, 2)	2 (1, 3)	3 (2, 4)	2 (1, 3)
Total treatment time (hours) (IQR)^c^	5.5 (0, 18.5)	6.5 (0, 17)	15.5 (9, 23)	10 (1.25, 20.5)

^a^Time to antivenom missing/unknown in *n* = 16 cases

^b^Number of administrations (excludes maintenance doses for CroFab)

^c^Time from first antivenom to last antivenom administration, missing in *n* = 13 cases

One hundred one (35%) patients received CroFab therapy only, 110 (38%) received Anavip therapy only, and 80 (27%) received both products. The median time from envenomation to a patient receiving their first vial of antivenom was 2.5 hours (IQR: 1.75, 4).

**Fig. 2:**
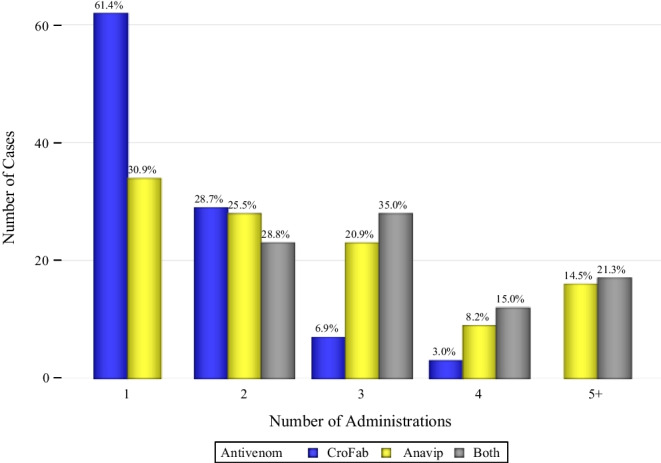
Number of administrations by antivenom type. Scheduled CroFab maintenance doses were considered part of the first administration.

After stepwise variable selection, the final model included antivenom type and envenomation location to predict the number of antivenom vials (Supplemental Table 1). CroFab was associated with fewer antivenom vials used (*p* < 0.0001). The median number of antivenom vials administered for those that received CroFab was 10 vials (IQR: 6, 12) and the cohort that received Anavip had a median of 18 vials (IQR: 10, 25). The median number of vials for patients that received both CroFab and Anavip was 20 vials (IQR: 16, 28). The median number of administrations was one (IQR: 1, 2) for CroFab, two (IQR: 1, 3) for Anavip, and three (IQR: 2, 4) for both antivenoms. Sixty-two (61%) patients that received CroFab only had one antivenom administration while only 34 (31%) patients that received Anavip had a single administration (Fig. 2). Scheduled maintenance doses were used in 47% of patients that received CroFab. The median total treatment time for each group was 5.5 hours (IQR: 0, 18.5), 6.5 hours (IQR: 0, 17), and 15.5 hours (IQR: 9, 23), for CroFab only, Anavip, and treatment of both products, respectively (Table 2).

**Table 2:** Outcomes by antivenom type.

Outcome variable	CroFab *n* **=** 101	Anavip *n* **= **110	Both antivenoms *n ***= **80	*P*-value*
Total number of vials				
Median (IQR)	10 (6, 12)	18 (10, 25)	20 (16, 28)	< 0.0001
Time to antivenom (hours)^a^				
Median (IQR)	2.0 (1.5, 4)	3.0 (1.5, 5)	2.5 (2, 3.5)	0.3961
Total treatment time (hours)^b^				
Median (IQR)	5.5 (0, 18.5)	6.5 (0, 17)	15.5 (9, 23)	< 0.0001
Number of administrations^c^				
Median (IQR)	1 (1, 2)	2 (1, 3)	3 (2, 4)	< 0.0001
Hospital length of stay (days)^d^				
Median (IQR)	2 (2, 2)	2 (2, 3)	2 (2, 3)	0.0093

*P-value from nonparametric Kruskal-Wallis test

^a^Time to antivenom missing/unknown in *n* = 16 cases

^b^Time from first antivenom to last antivenom administration, missing in *n* = 13 cases

^c^Number of administrations (excludes maintenance doses for CroFab)

^d^Total time in hospital missing for *n* = 1 case

The median number of days of hospital length of stay for each group was two days (IQR: 2, 2) for the CroFab group, two days (IQR: 2, 3) for the Anavip group, and two days (IQR: 2, 3) for those who received both antivenoms.

## Discussion

This study revealed that patients with rattlesnake envenomations received fewer vials and administrations of CroFab antivenom than those receiving Anavip. Patients were similar in demographics and envenomation symptoms between antivenom groups, though CroFab-treated patients had slightly more coagulopathy, neurotoxicity, and systemic symptoms. This is the first comparative multicenter analysis of dosing between the two antivenoms in the post-market landscape since Anavip was made commercially available for use in late 2018.

The cohort demographics were similar to prior snakebite studies in the USA [2, 3, 8]. Regionally, most reported envenomations from this dataset were from rattlesnakes in the Western USA (96%). This result is multifactorial. First, we have excluded *Agkistrodon* envenomations from this analysis since so few received Anavip and we hoped to analyze as homogenous a dataset as possible. The Eastern USA has multiple pit vipers, including copperheads, cottonmouths, and several species of rattlesnakes. In these regions, copperheads typically account for the largest number of envenomated patients [9]. Though, on average, a less potent envenomation, the clinical effects of copperheads can be indiscernible from a rattlesnake envenomation [9, 10]. This, combined with patients often having difficulty identifying venomous snake species, may have led to envenomations being coded as “unknown” or “other” snake types, and thus these cases were excluded from these analyses. Furthermore, the F(ab’)2 product was not FDA approved to treat *Agkistrodon* envenomations until April 2021 [6]. When faced with a choice to continue using an established antivenom or trying a new antivenom that is only FDA approved to treat a minority of envenomations in the region, a large majority of hospitals where both *Agkistrodon* and *Crotalus* envenomations occur likely opted to continue to use the CroFab antivenom. We postulate that this significantly contributed to why few patients from the Eastern states received F(ab’)2 products in this data set.

Clinical effects are consistent with prior studies of rattlesnake envenomations from the NASBR [2]. Every patient except one had local tissue damage recorded. Unsurprisingly, there were no deaths in the dataset, given the rarity of deaths from pit vipers in the USA [1, 2]. Snakebite envenomations have better outcomes when appropriate treatments are given earlier [11-13]. Total treatment time between the Fab and F(ab’)2 groups were similar, with those receiving both treatments having a much longer total treatment time. This is logical given that patients receiving both antivenoms likely had one of two scenarios occur. The first scenario is the initiation of treatment with one type of antivenom at an outside facility, followed by transfer to a larger facility where medical toxicology consultation was available, and treatment with the other antivenom. The second scenario was that patients were considered a “treatment failure” due to a lack or limited response to one antivenom, so the other antivenom was used to see if a better response would occur. The median number of total days in the hospital was similar with a median of 2 days, though the range was larger in the Anavip and both antivenom groups. However, this may not be clinically significant. Clearly, many factors affect treatment time, including time of presentation, patient age, and patient comfort/desire for discharge. Future studies should examine total hours in the hospital instead of days to assess this difference with additional attention to confounding factors.

While the mean number of total vials of antivenom was significantly different between the CroFab (10.19) and Anavip (19.98) groups (*p* < 0.0001), it is essential to note that the dosing regimens are different. The mean doses for each group are similar to other literature [7, 9, 14]. A 2011 study of CroFab used a median of 9 vials to achieve control [15], a study in 2011 found similar mean doses for CroFab [15], and the 2015 Phase 3 trial of Anavip used a mean of 16.1 vials [7]. Our data demonstrate that Crofab had fewer median administrations than Anavip (1 vs 2), similar to a prior single-center study [14]. Twice as many patients in the CroFab group received only a single administration course (61% versus 31%) compared to the Anavip group (Fig. 2). The reasons for additional administrations in the Anavip group are unclear and unexpected given the longer half-life of the F(ab’)2 product. Possible explanations include the administration of an insufficient neutralizing antivenom dose, lack of venom component match to North American snake venom in the Anavip product, or other treatment considerations not adequately captured by NASBR. One case series demonstrated that more patients were treated effectively for neurotoxicity with CroFab compared to Anavip, although only 7 patients were included in that series [16]. Clinical response frequently drives redosing decisions and some venom effects may respond better to one product over the other. Scheduled maintenance doses were used in 47% of patients that received CroFab alone in our dataset. It is unclear if those patients would have needed additional doses if no maintenance dosing was given. A prior study illustrated that scheduled maintenance dosing versus re-dosing as needed resulted in the same median total number of vials needed to treat the patient [12]. The benefit of scheduled maintenance dosing for limiting delayed thrombocytopenia remains unclear. Furthermore, the rate of hematologic recurrence varies greatly among rattlesnake species around the USA.

Due to these factors, there is no one size fits all approach to the dynamic process of treating snake envenomations. Medical toxicologists’ practice patterns vary on whether they give maintenance doses or re-administer antivenom boluses depending on the local envenomation syndromes, region-specific data, and anecdotal experience treating envenomation. Increased administration clearly has cost implications for envenomated patients and hospitals stocking antivenoms. These practices may be different in *Agkistrodon* envenomated patients. Additional work is needed to determine the reasons why more Anavip vials and administrations are administered compared to CroFab.

## Limitations

The severity of envenomation was not assessed in this analysis due to the lack of a standardized tool in the NASBR. The NASBR has inherent limitations due to the voluntary reporting of data to the ToxIC registry. Medical toxicologists submitting data to the NASBR are primarily located at academic tertiary care centers, which may not reflect how snakebites are managed in the community setting. Furthermore, some NASBR sites receive a disproportionally higher number of envenomations, which may skew the data from those sites’ nuanced practice patterns.

Most NASBR patients are seen at the time of presentation, and follow-up is often done outside of medical toxicology clinics. Lack of follow-up data limits the endpoints that can be analyzed using this data and adverse events, recurrence and other patient outcomes could not be assessed. While unlikely, it is possible that venom effect recurrence was not captured, and this may have resulted in additional antivenom administration in either group.

Additional investigation is warranted for patients who received both antivenoms as NASBR does not contain information on why the change in antivenom occurred and observations may be confounded by severity, occurrence of adverse reactions, delays in treatment, and transfers.

## Conclusion

Patients treated for rattlesnake envenomations by medical toxicologists participating in the NASBR received more antivenom vials and more administrations when Anavip was administered compared to CroFab. Further studies should assess if this same trend occurs in *Agkistrodon* envenomations.

## Supplementary Information

ESM 1: Supplemental Appendix (DOCX 20 kb)

ESM 2: Supplemental Table 1 (DOCX 23 kb)
