# Supplementary material for: Total CroFab and Anavip Antivenom Vial Administration in US Rattlesnake Envenomations: 2019–2021
Source: J Med Toxicol. 2023 Apr 28;19(3):248–54. doi: 10.1007/s13181-023-00941-7 (PMC10293487; doi:10.1007/s13181-023-00941-7)
Supplement: Supplementary file 1 — Supplemental Appendix (DOCX 20 kb) [file 13181_2023_941_MOESM1_ESM.docx]

**Supplemental Appendix:**

Western states included: Wyoming, Montana, Idaho, Washington, Oregon, California, Nevada, Utah, Colorado, New Mexico, and Arizona.

Eastern states included: North Dakota, South Dakota, Nebraska, Kansas, Oklahoma, Minnesota, Iowa, Missouri, Wisconsin, Illinois, Michigan, Indiana, Kentucky, Virginia, West Virginia, Ohio, Pennsylvania, Maryland, Delaware, New Jersey, New York, Kentucky, Rhode Island, Vermont, New Hampshire, Maine, Texas, Louisiana, Arkansas, Mississippi, Alabama, Georgia, Florida, Tennessee, North Carolina, South Carolina.
