# Supplementary material for: Total CroFab and Anavip Antivenom Vial Administration in US Rattlesnake Envenomations: 2019–2021
Source: J Med Toxicol. 2023 Apr 28;19(3):248–54. doi: 10.1007/s13181-023-00941-7 (PMC10293487; doi:10.1007/s13181-023-00941-7)
Supplement: Supplementary file 2 — Supplemental Table 1 (DOCX 23 kb) [file 13181_2023_941_MOESM2_ESM.docx]

**Supplemental Table 1.** Model parameters to predict total vials by antivenom used, after controlling for demographic and baseline characteristics

| **Demographic Variable** | ***Univariate***  ***p-value*** | ***Multivariate***  ***Full^a^***  ***p-value*** | ***Multivariate***  ***Initial Stepwise***  ***p-value*** | ***Multivariate***  ***Final Stepwise^b^***  ***P-value*** |
| --- | --- | --- | --- | --- |
| Age Category | 0.7289 | 0.1564 | - | - |
| Sex | 0.9410 | 0.5779 | - | - |
| Envenomation Location | 0.1198 | 0.1188 | 0.1507 | 0.1292 |
| Region | 0.0081 | 0.6607 | 0.6670 | - |
| Year | 0.0258 | 0.7788 | 0.3477 | - |
| Time to Antivenom | 0.6358 | 0.3961 | - | - |
| Antivenom type | <0.0001 | <0.0001 | <0.0001 | <0.0001 |
| **Parameter** | **Parameter Estimates from**  **Final Stepwise Model** | | |  |
|  | **Estimate** | **SE** |  |  |
| Base Case (Upper Extremity, Anavip) | 20.79 | 1.05 |  |  |
| Lower Extremity | -1.61 | 1.06 |  |  |
| Both | 2.72 | 1.32 |  |  |
| CroFab | -9.79 | 1.24 |  |  |
|  | **Mean^c^** | **SE** | **P-value** | **Pairwise P-value**  **(vs Anavip)** |
| **Antivenom type** |  |  | <0.0001 |  |
| Both | 22.71 | 1.00 |  | 0.0402 |
| CroFab | 10.19 | 0.89 |  | <0.0001 |
| Anavip | 19.98 | 0.86 |  | - |

^a^ Full model N=275 due to n=16 cases missing time to antivenom.

^b^ Final stepwise selection criteria, p<0.15 to enter model, p<0.15 to stay in model

^c^ Least squares mean estimated from final stepwise model
